# Postnatal Growth Restriction Is Reduced If Birth Weight Is Used for Nutritional Calculations in ELBW Infants

**DOI:** 10.1155/2018/2045370

**Published:** 2018-11-11

**Authors:** Pradeep Alur, Harithsa Asuri, Jane Cirelli, Ankita Patel, Theodore Bell, Jonathan Liss, Naveed Hussain

**Affiliations:** ^1^University of Mississippi Medical Center, Jackson, MS, USA; ^2^American University of Antigua College of Medicine, Antigua, Antigua and Barbuda; ^3^Pediatrics, WellSpan Health, York, PA, USA; ^4^University of Health Sciences, Farmington, CT, USA

## Abstract

Since fluid and nutrition needs and delivery in ELBW infants are calculated based on their body weights, there could be a measurable difference in fluid, nutrition, and protein intake calculations based on birth weight (BW) or current weight of the infant, especially in the first two weeks of life. Theoretically, the use of current daily weight (CW) for calculations may result in decreased fluid, nutrition, and protein delivery as well as a cumulative protein deficit (cPD) over the first two weeks of life until the infant regains birth weight. However, there have been no clinical studies comparing the clinical and nutritional impact of these two strategies is unknown.* Aims*. The aims of this study were to quantify the amount of protein intake and to compare growth parameters at hospital discharge (as measured by discharge weight and head circumference percentiles) when using two different methodologies (BW vesrsus current daily weight until BW is regained) for calculating fluid and protein intake in the first two weeks after birth in ELBW infants.* Methods*. A retrospective review of infants weighing ≤ 1kg at birth was conducted from January 2005 to December 2009 (Phase 1; P1) and January 2012 to December 2014 (Phase 2; P2) in a tertiary care NICU. At this center, in P1 (2005-09) CW was exclusively used for calculating fluid, calorie, and protein administration till BW was regained. In P2 (2012-14), BW was exclusively used for all calculations. Both P1 and P2 periods were compared and analyzed for differences in demographics, nutritional intake, comorbid conditions, and growth outcomes.* Results*. We studied 146 infants with 84 and 62 infants in P1 and P2 periods, respectively. The mean gestational age was lower during Phase 1 (25.74 ±1.32 vs. 26.47 ±1.82 weeks. P value =0.01). However, the birth weights were not different between the two periods. When the multiple-regression analysis was done using a discharge weight of >10^th^ percentile as the dependent variable, protein intake before regaining of BW (OR of 4.126 with 95^th^ CI of 2.03-8.36, a P value of 0.00) and AGA status at birth (OR of 8.37 with 95^th^ CI of 2.67-26.24) remained significant factors. Compared to P1, babies in P2 received 1g/kg/day more protein till BW was regained.** In P1, 27**% of babies who were appropriate for gestational age (AGA) for head circumference at birth became** microcephalic** by discharge,** compared** to** 15.6**%** in P2 **(p=0.03). Similarly,** 75.3**%** of the babies who were AGA for weight at birth in P1 became small for gestational age (SGA) by discharge**,** compared to 16.7**%** in P2 **(p=<0.0001). The number of days it took to regain BW was 9.6 days in P1 vs. 7 days in P2 (p=<0.0001).* Conclusions*. Basing nutrition calculations in ELBW on birth weight rather than current daily weight until the birth weight is regained resulted in significantly greater protein delivery, a significant decrease in the incidence of failure to thrive and smaller head circumference percentiles at discharge in ELBW infants.

## 1. Background

During the 2nd and early 3rd trimester, fetuses receive about 4+/- 0.5gr/kg/day of protein [1]. A study from NICHD showed that >90% ELBW (extremely low birth weight) infants were growth restricted by 36 weeks of post-menstrual gestational age (PMA) [2]. This prompted the promotion of early aggressive nutrition in this vulnerable population [3]. Protein delivery increases steadily as the fetus grows and gains weight in utero. In trying to replicate this high protein intake, studies have shown that institution of 3gm/kg/day of protein on day 1 of life was safe [4]. It is also recognized that neonatal brain growth is easily compromised during the first few weeks of life. Hence, early protein deficits may be linked to poor neurodevelopmental and growth outcomes [5]. Moreover, recent studies have shown a direct association between first-week nutritional intakes, especially higher protein intake, with improved neurodevelopment at 18-24 months [6, 7]. Based on information from such studies, the use of 3-4gm/kg/day of protein in parenteral nutrition for ELBW infants within the first few days of life has become the standard goal.

In the premature infant, growth and weight gain do not always occur in the first few days or weeks after birth. An ELBW infant may lose 10-20% of its birth weight (BW) and may take 2 to 3 weeks to regain its BW. Since fluid and nutrition need and delivery in ELBW infants are calculated based on their body weights, there could be a measurable difference in fluid, nutrition, and protein intake calculations based on birth weight or current weight of the infant, especially in the first two weeks of life. The issue is further complicated by the fact that fluid overload in the early newborn period is associated with negative cardiorespiratory outcomes, and most centers pay close attention to the daily fluctuations in weight when managing fluid balance in these infants [8]. Theoretically, the use of current daily weight (CW) for calculations may result in decreased fluid, nutrition, and protein delivery, as well as a cumulative protein deficit (cPD) over the first two weeks of life until the infant regains birth weight. However, there have been no clinical studies comparing the targeted delivery with the actual delivery of nutrients and protein using these two methodologies, and the clinical and nutritional impact of these two strategies is unknown. At our NICU, a change in methodology for the calculation of fluids and nutrition from CW to BW was made in 2012 and offered a clinical opportunity for a comparative study.

The aims of this study were to quantify the amount of protein intake and compare growth parameters at hospital discharge (as measured by discharge weight and head circumference percentiles) when using two different methodologies (birth weight versus current daily weight until birth weight is regained) for calculating fluid and protein intake in the first two weeks after birth in ELBW infants.

## 2. Methods

A retrospective review of infants weighing ≤ 1kg at birth was conducted from January 2005 to December 2009 (Phase 1; P1) and January 2012 to December 2014 (Phase 2; P2) in a tertiary care NICU. All ELBW admissions to the NICU were included except those regaining BW in ≤ 3 days, those who were transferred to another facility, or those who died before reaching discharge. Birth weight, current daily weight, caloric intake (Kcal/kg/day), total fluid intake (mL/kg/day), and parenteral protein intake (gm/kg/day) were recorded daily until BW was regained and until discharge. At this center, in P1 (2005-09) CW was exclusively used for calculating fluid, calorie, and protein administration till BW was regained. In P2 (2012-14), BW was exclusively used for all calculations. This study was initiated as a patient care improvement project in 2010, and recommendations were made in 2011. Hence, the follow-up phase of the project was started in 2012.

Comorbid conditions that may influence an infant's nutritional needs and growth outcomes were studied. CLD (chronic lung disease) was defined as the requirement for oxygen at 36 weeks of corrected gestational age [9]. PDA (patent ductus arteriosus) was defined as the presence of patent ductus arteriosus on an echocardiogram. NEC (necrotizing enterocolitis) was defined as the presence of pneumatosis intestinalis or air in the portal vein on an X-ray or surgical diagnosis at the time of laparotomy [10]. IVH (intraventricular hemorrhage) was defined as the presence of any hemorrhage in the lateral ventricles of the brain on an ultrasound [11]. Gender-specific Fenton growth charts (2013) were used for growth assessment [12]. The length was not analyzed due to intrinsic variability in measurement secondary to lack of standard length board. This study received institutional review board exemption as part of the quality improvement.

Feeding and nutritional practices during the two phases were as follows.

In P1 (2005-09), infants were gavage-fed ≤15ml/kg/day for seven days. Soon after birth, protein-based early parenteral nutrition was initiated with infants being given 2g/kg/day within 24 hours of birth. Fluids are initially started at 80-100 ml/kg/day and advanced as needed based on serum sodium levels. Enteral nutrition is calculated based on formula nutrient composition and average breast milk composition. Protein intake was calculated to provide 3-4 g/kg/day in the first week of life based on the current weight. Subsequently, daily administration of a protein of 3-4g/kg/day, calories of 120-130 kcal/kg/day, and fluids of 135-155 ml/kg/day was targeted based on CW, unless an infant's condition warranted a change. Human milk fortifier was used to fortify the breast milk to 24 cal/oz. Average targeted daily weight gain was 15-20 grams/kg/d. No probiotics were administered.

P2 (2012-14) infants were gavage-fed ≤15ml/kg/day for five days. Soon after birth, protein-based early parenteral nutrition was initiated with infants being given 2g/kg/day within 24 hours of birth. Protein intake was calculated to provide 3-4g/kg/day after 24 hours of age. Subsequently, daily administration of a protein of 3-4g/kg/day, calories of 120-130 kcal/kg/day, and fluids of 135-155 ml/kg/day was targeted based on BW, unless infant's condition warranted a change. Human milk fortifier was used to fortify the breast milk to 24 cal/oz. Average targeted daily weight gain was 15-20 grams/kg/d. Infants received a daily dose of probiotics (UDO's choice infant's probiotic, Flora Inc., Lynden, Washington, USA).

P1 and P2 periods were compared and analyzed for differences in demographics, nutritional intake, comorbid conditions, and growth outcomes.

Statistical analysis was completed using SPSS v.18. Descriptive statistics were summarized using frequencies and means. Wilcoxon Signed-rank test and Chi-square analyses were used to determine associations between variables.

## 3. Results

### 3.1. Demographics

We studied 146 infants with 84 and 62 infants in P1 and P2 periods, respectively. The demographics in both periods are given in Table 1. Mean gestational age was lower during Phase 1 (25.74 ±1.32 vs. 26.47 ±1.82 weeks. P value =0.01). However, the birth weights were not different between the two periods. The incidence of SGA at birth was higher during Phase 2. When the multiple-regression analysis was done using discharge weight of >10^th^ percentile as the dependent variable, protein intake before regaining of BW (OR of 4.126 with 95^th^ CI of 2.03-8.36, a P value of 0.00) and AGA status at birth (OR of 8.37 with 95^th^ CI of 2.67-26.24) remained significant factors.

### 3.2. Nutritional Intake before Regaining BW during P1 and P2

Nutritional intake comparisons between the two periods of the study are shown in Table 2. Compared to P1, babies in P2 received 1 g/kg/day more protein till BW was regained. This average daily protein deficit would have resulted in a cumulative protein deficit of 5.1 g/kg if the infant's current weight was used for protein calculations for the duration of 1 week (P2 phase) (see example in Table 3).

### 3.3. Nutritional Intake after Regaining BW during P1 and P2

Table 4 shows nutrient intakes between P1 and P2 periods after regaining of birth weight. There were no significant differences in protein and fluid intakes. However, there was a higher caloric intake in P2 compared to P1.

#### 3.3.1. Growth Outcomes

There were better growth outcomes for weight and head circumference in period 2 when current daily weight was used to calculate protein intake. The incidence of small for gestation at discharge irrespective of the birth-weight category was 78.5% in P1 and 43.5% in P2 (P = <0.001). However, In P1, 27% of babies who were appropriate for gestational age (AGA) for head circumference at birth became microcephalic by discharge, compared to 15.6% in P2 (p=0.03). Additionally, 75.3% of those AGA for weight at birth in P1 became small for gestational age (SGA) by discharge, compared to 16.7% in P2 (p=<0.0001). The number of days it took the infants to regain BW was 9.6 days in P1 vs. 7 days in P2 (p=<0.0001).

#### 3.3.2. Other Morbidities

There were no significant differences in other morbidities evaluated, specifically CLD, PDA, IVH, and NEC between the two periods of study (Table 5).

In the regression analysis for the predictors of discharge weight centiles below the 10th percentile for those who were AGA at birth showed that protein received during the first weeks until the regaining of birth weight was very significant with an odds ratio of 4.175 (95% CI 1.886-9.243) and a P value of <0.001. The number of calories delivered before regaining of birth weight (P = 0.995. OR-1. 95% CI = 0.965-1.036), NEC (P = 0.07. OR- 0.119. 95% CI = 0.012-1.165), and PDA (P value = 0.79. OR 0.89. 95% CI = 0.37-2.139) were not significant.

## 4. Discussion

The use of birth weight (BW) rather than current daily weight (CW) in calculating fluid and nutritional intakes during the initial part of an infant's stay in the NICU leads to significantly higher protein intake and is associated with higher percentiles achieved for weight and head circumference at the time of discharge. We believe that this simple change in NICU practice may have significant benefits for ELBW infants.

It has been shown that, just in the course of routine NICU care, preterm infants of ≤30 weeks could accumulate up to 14+/-3 g/kg of protein deficit in the first week of life [13]. Senterre indicated that, despite providing 96% of the recommended energy in the first weeks of life, the cumulative protein deficit in the first week of life remained a major determinant of the postnatal growth during first 6 weeks of life [14]. This cumulative protein deficit may not be adequately compensated in the course of the infant's NICU stay and may lead to adverse growth outcomes by the time of discharge. Franz et al. showed that improving early neonatal growth may improve long-term neurodevelopmental outcomes [15].

When using CW for nutritional calculations, we found that there was an average of a 2 gm/kg deficit of protein in the first week of life with some infants losing up to a 6.9 gm/kg during P1. It is possible that this protein deficit may not have been adequately compensated for, which led to significantly poorer weight and head circumference growth during P1 compared to P2. This may have an impact on future neurodevelopmental outcomes in these infants. Our findings may be corroborated by a recent study that showed that, for each gram/kg increase in protein delivery in the first week, there was an increase of 8.2 points in MDI (mental development index) [16]. Another study has shown that higher protein and calorie intake reduced postnatal head growth failure in preterm infants of <29 weeks' gestation [17]. Thus, using BW for protein calculations (till BW is regained) may be a simple, but effective, strategy to avoid significant protein deficits and consequent postnatal growth restriction. It is also important to consider when the additional protein is provided, as that may also be critical to avoid a fall in growth percentile. A study involving 560 children showed that growth during early infancy (0-4 months) predicted IQ at nine years of age [18].

Another aspect of neonatal physiology that impacts this issue is that a fetus in utero receives increasing amounts of protein every day, which accounts for its continuously increasing growth with advancing gestation. In contrast, soon after birth, this increasing protein transfer is not accounted for in the infant's nutrition, and the infant loses weight and may become catabolic. It may take up to a week or longer to regain BW and reverse the catabolic changes. During this period, even in the best circumstances, the infant would receive significantly less protein and other nutrients than it would have in utero. Therefore, there is a need for an additional allowance of nutritional requirements for catch-up growth [19].

The practice of using current daily weight (CW) for nutritional calculations does not appear to have any physiological basis. The concern for fluid overload and poor respiratory outcomes as raised by Van Mater et al. [8] may not be applicable in this instance because calculating fluid based on the baby's existing fluid compartment cannot be construed as “fluid overload.” We have observed that using birth weight for protein calculations (till birth weight was regained), instead of current daily weight, could result in improved growth outcomes in ELBW infants without any adverse effects on other neonatal morbidities.

A limitation of our study is that it is retrospective, and, hence, all confounders such as use of antenatal steroids, chorioamnionitis, hemodynamically significant PDA, and probiotics cannot be controlled. However, PDA, NEC, and calories were not significant in the regression analysis for AGA infants developing postnatal growth restriction. Though we did not detect a difference in the incidence of NEC, the study was not, however, powered to detect it. We have not evaluated long-term developmental/neurological outcomes in this cohort. Therefore, it cannot be inferred that our practice change had a long-term positive impact on neurological outcomes. However, the use of birth weight for calculating protein provision in the first few days to a week of life may significantly reduce the postnatal growth restriction and microcephaly in preterm infants weighing ≤ 1kg at birth. Multiple logistic regression analysis confirmed that protein administration before the regain of birth weight could significantly affect discharge weight percentiles.

## 5. Conclusion

Basing nutrition calculations in ELBW on birth weight rather than current daily weight until the birth weight is regained resulted in significantly higher protein delivery, a significant decrease in the incidence of failure to thrive, and smaller head circumference percentiles at discharge in ELBW infants. The findings from this single-center retrospective study with historical controls need to be corroborated with a larger multicenter population of ELBW infants to confirm the above conclusion.

## Figures and Tables

**Table 1:** Patient characteristics.

	Phase 1 (2005-09) (N=84). P1		Phase 2 (2012-14) (N=62) P2.		
	Count	%	Count	%	Significance
**Female**	37	44.05%	31	52.54%	0.32
**Gest Age-mean, std dev**	25.74	1.32	26.47	1.82	0.01
**Birth Wt-mean, std dev**	766.98	127.95	744.84	166.82	0.37
**Birth Weight <10**%	11	13.10%	26	41.94%	<.001
**Birth HC <10**%	14	17.50%	17	27.42%	0.16
**Discharge Weight <10**%	66	78.57%	27	43.55%	<.001
**Discharge HC <10**%	27	33.33%	17	27.42%	0.45

**Table 2:** Daily nutritional intake before regaining birth weight.

	Phase 1 (2005-09) (N=84)		Phase 2 (2012-2014) (N=62)		
	Mean	SD	Mean	SD	Significance
**Protein Received**	2.37	0.44	3.37	0.38	0
**Calories Received**	62.98	12.9	67.06	13.39	0.07
**Fluids Received**	132.48	14.61	119.9	13.33	0

**Table 3:** An example of an infant receiving 4 g/kg protein based on daily weight and birth weight of an actual infant.

**DOL**	**1**	**2**	**3**	**4**	**5**	**6**	**7**	**8**	**9**	**10**	**11**	**12**	**13**	**14**
**BW**	0.78	0.78	0.78	0.78	0.78	0.78	0.78	0.78	0.78	0.78	0.78	0.78	0.78	0.78
**Protein based on BW**	3.12	3.12	3.12	3.12	3.12	3.12	3.12	3.12	3.12	3.12	3.12	3.12	3.12	3.12
**Current Daily Weight**	0.78	0.78	0.728	0.728	0.668	0.668	0.637	0.613	0.649	0.702	0.7	0.77	0.74	0.76
**Protein based on current daily weight**	3.12	3.12	2.91	2.91	2.67	2.67	2.55	2.45	2.60	2.81	2.80	3.08	2.96	3.04
**Protein deficit**	0	0	0.21	0.21	0.45	0.45	0.57	0.67	0.52	0.31	0.32	0.04	0.16	0.08

Cumulative protein given based on daily weight = 39.69 grams.

Cumulative protein given if birth weight = 43.68 gms.

Cumulative protein deficit = 3.99 grams (which is 5.1 g/kg protein deficit).

**Table 4:** Nutritional intake after regaining birth weight to the time of discharge.

**Parameter**	**Period**	**Mean**	**Std. Dev**	**Significance (P value)**
**Protein g/kg/day**	2005-09	3.23	0.32	0.131
	2012-14	3.36	0.36	
**Calories kg/day**	2005-09	110.12	6.41	0.035
	2012-14	113.3	6.01	
**Fluids cc/kg/day**	2005-09	143.04	10.38	0.117
	2012-14	139.53	8.1	

**Table 5:** Morbidity before hospital discharge between the two periods of study.

	Phase 1 (2005-09) (N=84). P1		Phase 2 (2012-2014) (N=62) P2.		
	Count	%	Count	%	Significance
**CLD**	30	35.71%	30	48.39%	0.12
**PDA**	52	61.90%	29	46.77%	0.07
**IVH**	16	19.05%	17	27.42%	0.23
**NEC**	9	10.71%	3	4.80%	0.345

## Data Availability

The patient nutritional data used to support the findings of this study may be released upon application to the INSTITUTIONAL REVIEW BOARD of WellSpan Health that can be contacted through Dr. Theodore Bell: tbell@wellspan.org.

## References

[1] Hay W. W., Lucas A., Heird W. C. (1999). Workshop summary: Nutrition of the extremely low birth weight infant. *Pediatrics*.

[2] Ehrenkranz R. A., Younes N., Lemons J. A. (1999). Longitudinal growth of hospitalized very low birth weight infants. *Pediatrics*.

[3] Thureen P. J., Hay W. W. (2001). Early aggressive nutrition in preterm infants. *Seminars in Fetal and Neonatal Medicine*.

[4] Khanam S., Khan J., Sharma D., Chawla D., Murki S. (2015). Nutritional bundle to improve growth outcomes among very low birth weight infants. *The Journal of Maternal-Fetal and Neonatal Medicine*.

[5] Lucas A., Morley R., Cole T. J. (1998). Randomised trial of early diet in preterm babies and later intelligence quotient. *British Medical Journal*.

[6] Yang J., Chang S. S. Y., Poon W. B. (2016). Relationship between amino acid and energy intake and long-term growth and neurodevelopmental outcomes in very low birth weight infants. *Journal of Parenteral and Enteral Nutrition*.

[7] Cormack B. E., Bloomfield F. H., Dezoete A., Kuschel C. A. (2011). Does more protein in the first week of life change outcomes for very low birthweight babies?. *Journal of Paediatrics and Child Health*.

[8] Marter L. J. V., Leviton A., Allred E. N., Pagano M., Kuban K. C. K. (1990). Hydration during the first days of life and the risk of bronchopulmonary dysplasia in low birth weight infants. *Journal of Pediatrics*.

[9] Shennan A. T., Dunn M. S., Ohlsson A., Lennox K., Hoskins E. M. (1988). Abnormal pulmonary outcomes in premature infants: Prediction from oxygen requirement in the neonatal period. *Pediatrics*.

[10] Bell M. J., Ternberg J. L., Feigin R. D. (1978). Neonatal necrotizing enterocolitis. Therapeutic decisions based upon clinical staging. *Annals of Surgery*.

[11] Papile L. A., Burstein J., Burstein R., Koffler H. (1978). Incidence and evolution of subependymal and intraventricular hemorrhage: a study of infants with birth weights less than 1,500 gm. *Journal of Pediatrics*.

[12] Fenton T. R., Nasser R., Eliasziw M., Kim J. H., Bilan D., Sauve R. (2013). Validating the weight gain of preterm infants between the reference growth curve of the fetus and the term infant. *BMC Pediatrics*.

[13] Embleton N. E., Pang N., Cooke R. J. (2001). Postnatal malnutrition and growth retardation: An inevitable consequence of current recommendations in preterm infants?. *Pediatrics*.

[14] Senterre T., Rigo J. (2012). Reduction in postnatal cumulative nutritional deficit and improvement of growth in extremely preterm infants. *Acta Paediatrica*.

[15] Franz A. R., Pohlandt F., Bode H. (2009). Intrauterine, early neonatal, and postdischarge growth and neurodevelopmental outcome at 5.4 years in extremely preterm infants after intensive neonatal nutritional support. *Pediatrics*.

[16] Stephens B. E., Walden R. V., Gargus R. A. (2009). First-week protein and energy intakes are associated with 18-month developmental outcomes in extremely low birth weight infants. *Pediatrics*.

[17] Morgan C., McGowan P., Herwitker S., Hart A. E., Turner M. A. (2014). Postnatal head growth in preterm infants: A randomized controlled parenteral nutrition study. *Pediatrics*.

[18] Pongcharoen T., Ramakrishnan U., Di Girolamo A. M. (2012). Influence of prenatal and postnatal growth on intellectual functioning in school-aged children. *JAMA Pediatrics*.

[19] Adamkin D. H. (2006). Nutrition management of the very low-birthweight infant: ii. optimizing enteral nutrition and postdischarge nutrition. *NeoReviews*.

